# Mapping of the binding sites of human histamine *N*-methyltransferase (HNMT) monoclonal antibodies

**DOI:** 10.1007/s00011-017-1086-7

**Published:** 2017-08-08

**Authors:** Hubert G. Schwelberger, Johannes Feurle, Gunnar Houen

**Affiliations:** 1 Molecular Biology Laboratory, Department of Visceral, Transplant and Thoracic Surgery, Medical University Innsbruck, Schöpfstraße 41, 6020 Innsbruck, Austria; 2 Department of Autoimmunology and Biomarkers, Statens Serum Institut, Artillerivej 5, 2300 Copenhagen, Denmark

**Keywords:** Histamine *N*-methyltransferase, Histamine metabolism, Monoclonal antibodies, Protein expression, Epitope mapping

## Abstract

**Objective:**

Recently, we characterized mouse monoclonal antibodies that allow the specific and sensitive detection of human histamine *N*-methyltransferase (HNMT). To understand differences in binding characteristics and recognition of enzyme variants we mapped the antibody binding sites.

**Methods:**

Fragments of human HNMT were expressed as glutathione *S*-transferase fusion proteins that were used for testing antibody binding on immunoblots. Combined information from species cross-reactivity, sequence comparison, protein structure, and binding site prediction software were used to localize the epitope recognized by each antibody.

**Results:**

All eight monoclonal HNMT antibodies bound to linear epitopes in the C-terminal domain of the 292 amino acid protein. Of the five antibodies cross-reacting with HNMT from other species, one bound region L^182^–T^223^, three region M^224^–E^261^, and one region L^262^–A^292^. All three antibodies recognising only human HNMT bound the C-terminal region L^262^–A^292^ that contains residues present only in the human protein.

**Conclusions:**

Our HNMT monoclonal antibodies bind in three different regions of the protein and those binding the same putative epitope exhibit similar binding characteristics and species cross-reactivity. Antibodies binding non-overlapping epitopes will facilitate analyses of all clinically relevant variants described for HNMT.

## Introduction

Histamine binds and activates four different G-protein-coupled receptors and thereby mediates many biological processes including inflammation, gastric acid secretion, neuromodulation, and regulation of immune function [1, 2]. Histamine exhibits pharmacological activity at very low concentrations, and therefore, its synthesis, transport, storage, release and degradation have to be tightly regulated to avoid undesirable reactions. Histamine synthesis is catalyzed by the enzyme histidine decarboxylase (HDC, EC 4.1.1.22) through decarboxylation of the amino acid l-histidine [3, 4]. In mammals, histamine can be inactivated either by oxidative deamination of the primary amino group, catalyzed by diamine oxidase (DAO, EC 1.4.3.22), or by methylation of the imidazole ring, catalyzed by histamine *N*-methyltransferase (HNMT, EC 2.1.1.8) [4–6].

Human HNMT is a small monomeric protein of 33 kDa consisting of a single polypeptide chain of 292 amino acid residues and catalyzes the transfer of a methyl group from *S*-adenosyl-l-methionine (SAM) to the secondary amino group of the imidazole ring of histamine forming *N* ^τ^-methylhistamine [6]. The human protein is encoded by a single gene designated HNMT that has six exons and has been mapped to chromosome 2q22.1 [7]. HNMT has a two-domain structure with the larger N-terminal domain being a classic methyltransferase fold with an SAM binding motif [8]. HNMT exhibits high substrate specificity for histamine and is inhibited by its reaction products, as well as by the SH-group reagents *p*-chloromercuribenzoate and *N*-ethylmaleimide and by the antimalarial drugs quinacrine and amodiaquine [6]. HNMT is a cytosolic protein that is responsible for the inactivation of intracellular histamine, which is either synthesized in the cell or taken up from the extracellular space after binding to one of its receptors present on the cell surface or by plasma membrane transporters [2, 4].

To characterize the human histamine-inactivating enzymes, we recently produced and characterized a series of mouse monoclonal antibodies specific for DAO and HNMT, respectively [9, 10]. These antibodies turned out to be invaluable tools for the study of the expression and cellular localization of the enzymes. The eight monoclonal antibodies binding human HNMT exhibited considerable differences in their binding characteristics and species cross-reactivity [10]. Additionally, we wanted to know if our antibodies recognize and can be used to detect the enzyme variants that have been described resulting from single nucleotide polymorphisms (SNPs) of the HNMT gene and that might be relevant for diseases associated with impaired histamine inactivation [11, 12]. Therefore, we tested antibody binding to various fragments of the HNMT protein expressed in vitro and combined these results with data from sequence comparison, species cross-reactivity, structural information, and binding site prediction tools to map the epitopes recognized by the different HNMT antibodies.

## Materials and methods

### Preparation and expression of recombinant human HNMT protein fragments

**Fig. 1:**
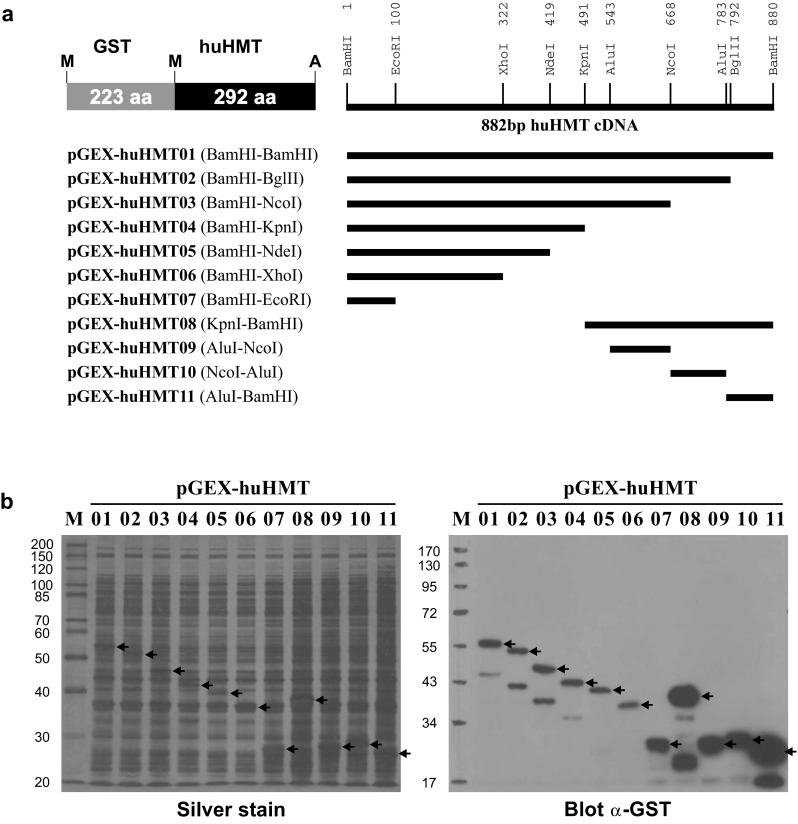
HNMT expression constructs. **a** Recombinant plasmids expressing GST-HNMT fusion proteins obtained by cloning different human HNMT cDNA fragments into the bacterial expression vectors pGEX-2T or pGEX-5X-1/-2, respectively. **b** 10% silver-stained SDS polyacrylamide gel and immunoblot with an anti-GST antibody of lysates prepared from bacteria harbouring plasmids pGEX-huHMT01-11 indicated on *top of each lane*. Migration positions of full-length fusion proteins are indicated by *arrows* and their expected sizes are listed in Table 1. The sizes of molecular weight markers (*M*) are given on the *left* in kDa

Full-length human HNMT cDNA [13, 14] was amplified by PCR with specific primers from total human kidney cDNA and cloned in frame into the *Bam*HI site of the bacterial expression vector pGEX-2T (GE Healthcare, Vienna, Austria) to obtain plasmid pGEX-huHMT01 [10]. A series of C-terminal deletions of HNMT was produced by double-digestion of pGEX-huHMT01 with restriction endonucleases *Eco*RI^100^, *Xho*I^322^, *Nde*I^419^, *Kpn*I^491^, *Nco*I^668^, or *Bgl*II^792^ (superscripts indicate position of recognition sequence relative to A of translational start codon) that cut once on the cDNA plus *Sma*I that cuts the vector immediately downstream of the cloning site, creating blunt ends by incubation for 15 min at 37 °C with 1 U Klenow Fragment and 100 µM dNTPs, and religating the respective larger fragments with T4 DNA ligase, resulting in plasmids pGEX-huHMT02-07. Fragments of the C-terminal region of HNMT were produced by digesting the 882 bp *Bam*HI fragment of full-length HNMT cDNA with *Kpn*I or *Alu*I + *Nco*I, creating blunt ends with Klenow Fragment, and ligating the gel-purified fragments *Kpn*I^491^–*Bam*HI^880^, *Alu*I^543^–*Nco*I^668^, *Nco*I^668^–*Alu*I^783^, and *Alu*I^783^–*Bam*HI^880^ in frame into the *Sma*I site of pGEX-5X-1 or pGEX-5X-2, respectively (GE Healthcare, Vienna, Austria), resulting in plasmids pGEX-huHMT08-11. All cloning enzymes were obtained from Thermo Scientific (Vienna, Austria). The clones were checked by DNA sequence analyses and their inserts and the resulting HNMT protein fragments are illustrated in Fig. 1a and detailed in Table 1.

**Table 1:** Expression plasmids for huHNMT fragments

Plasmid	Vector	cDNA fragment	Peptide	FuP (kDa)
pGEX-huHMT01	pGEX-2T	*Bam*HI^1^–*Bam*HI^880^	M^1^–A^292^	56.1
pGEX-huHMT02	pGEX-2T	*Bam*HI^1^–*Bgl*II^792^	M^1^–K^264^	53.0
pGEX-huHMT03	pGEX-2T	*Bam*HI^1^–*Nco*I^668^	M^1^–T^223^	48.5
pGEX-huHMT04	pGEX-2T	*Bam*HI^1^–*Kpn*I^491^	M^1^–T^164^	42.0
pGEX-huHMT05	pGEX-2T	*Bam*HI^1^–*Nde*I^419^	M^1^–H^140^	39.4
pGEX-huHMT06	pGEX-2T	*Bam*HI^1^–*Xho*I^322^	M^1^–N^107^	35.8
pGEX-huHMT07	pGEX-2T	*Bam*HI^1^–*Eco*RI^100^	M^1^–Q^33^	27.6
pGEX-huHMT08	pGEX-5X-1	*Kpn*I^491^–*Bam*HI^880^	N^165^–A^292^	38.1
pGEX-huHMT09	pGEX-5X-2	*Alu*I^543^–*Nco*I^668^	L^182^–T^223^	28.6
pGEX-huHMT10	pGEX-5X-1	*Nco*I^668^–*Alu*I^783^	M^224^–E^261^	29.2
pGEX-huHMT11	pGEX-5X-2	*Alu*I^783^–*Bam*HI^880^	L^262^–A^292^	27.4

Human HNMT cDNA fragments obtained with different restriction endonucleases were cloned in frame into the expression vectors pGEX-2T or pGEX-5X-1/-2 to produce different size GST-HNMT fusion proteins (FuP). Superscripts indicate position of restriction site on cDNA sequence and amino acid position, respectively

Each recombinant plasmid was transformed into the protease-deficient strain *E. coli* BL21 to produce glutathione *S*-transferase (GST) fusion proteins according to the manufacturer’s instructions (GE Healthcare, Vienna, Austria). Briefly, recombinant bacteria were grown at 37 °C with slight agitation (100 rpm) in 10 ml YTA (16 g/l tryptone, 10 g/l yeast extract, 5 g/l NaCl, 100 mg/l ampicillin, pH 7.0) to an OD_600nm_ of 0.5 and fusion protein expression was induced for 4 h by addition of 0.1 mM isopropyl-β-d-thiogalactopyranoside (IPTG, Roche, Vienna, Austria). Bacteria were harvested by centrifugation for 5 min at 4000×*g*, 4 °C, washed with cold deionized water, and lysed in SDS sample buffer by incubation for 10 min at 95 °C. Cell lysates were cleared by centrifugation for 5 min at 10,000×*g* and stored at −20 °C until use. Fusion protein expression was analyzed by SDS polyacrylamide gel electrophoresis [15] and Western blotting [16] using the GST-specific monoclonal antibody HYB374-01, which showed considerable expression for all constructs (Fig. 1b).

### Testing of the binding of HNMT specific monoclonal antibodies

Monoclonal antibodies HYB372-04/-05/-06/-07/-08/-09 and HYB373-02/-03 specific for human HNMT [10] were tested for binding to different HNMT fragments using filter strips of cell lysates containing the expressed GST fusion proteins. Cleared cell lysates prepared in SDS sample buffer containing approximately 100 µg of protein were separated on 12.5% SDS polyacrylamide gels [15] and blotted onto polyvinylidene fluoride (PVDF) membranes [16]. After washing in TBST (50 mM Tris·HCl, pH 7.5, 150 mM NaCl, 0.1% Tween 20) and blocking non-specific binding sites by incubation for 60 min at 4 °C in TBSTM (TBST containing 2% non-fat dry milk) the membranes were cut into 20 vertical filter strips each containing circa 5 µg of protein. Each filter strip was incubated for 16 h at 4 °C with suitable dilutions of the monoclonal antibodies in TBSTM, washed 4 × 5 min with TBST, incubated for 60 min at 4 °C with horseradish peroxidase-conjugated anti-mouse immunoglobulins (Dako, Glostrup, Denmark) diluted 1:1500 in TBSTM, washed for 4 × 5 min with TBST, incubated for 5 min with ECL reagent (GE Healthcare, Vienna, Austria), and exposed to Cronex 5 film (Agfa, Mortsel, Belgium).

Immunoprecipitation experiments were carried out to test if the antibodies also bind the native HNMT protein. Total lysates were prepared from human and porcine kidney and liver, respectively, by homogenization of ca. 50 mg tissue in 1 ml lysis buffer (20 mM bis·Tris·HCl, pH 7.0, 5 mM dithiothreitol) containing Complete Protease Inhibitor Cocktail (Roche, Vienna, Austria) for 5 min at 30 Hz using a TissueLyser II homogenizer (Qiagen, Hilden, Germany). Lysates were cleared by centrifugation for 10 min at 20,000×*g*, 4 °C and the supernatant containing the total soluble protein was stored at −20 °C until used. Lysates containing comparable HNMT activity were incubated with slight agitation in a total volume of 100 µl with different concentrations of the monoclonal HNMT antibodies for 16 h at 4 °C, followed by incubation with Protein A-Sepharose (GE Healthcare, Vienna, Austria) for 1 h at 4 °C. Immunoprecipitates were separated by centrifugation for 1 min at 6700×*g*, 4 °C, washed three times with TBST, and solubilized in SDS sample buffer. The presence of HNMT was analyzed in the precipitate and in the supernatant by immunoblotting with HYB372-07 and HNMT activity was determined in the supernatant by methylation of histamine with *S*-adenosyl-l-[methyl-^14^C]methionine (GE Healthcare, Vienna, Austria) as described previously [17]. Precipitation with a non-specific monoclonal antibody served as control.

### Mapping of binding sites using binding information, sequence comparison, and antibody cross-reactivity

HNMT polypeptide sequences were aligned using the NCBI constrained-based Multiple Alignment Tool (http://www.ncbi.nlm.nih.gov/tools/cobalt/cobalt.cgi?link_loc=BlastHomeLink) [18]. Antigenicity plots were produced with the BepiPred Linear Epitope Prediction Tool (tools.immuneepitope.org/bcell) [19]. For testing species cross-reactivity, filter strips prepared from cleared tissue lysates of human, porcine, rat, and mouse kidney were incubated with different HNMT antibodies and developed as described above. For detection of weak bands, ECL Prime reagent (GE Healthcare, Vienna, Austria) was substituted for ECL reagent. Structural views were created with the NCBI Cn3D 4.3.1 software [20] using HNMT structure 1JQD [8].

## Results

Using human and porcine HNMT expressed in vitro as antigens, we recently produced a series of mouse monoclonal antibodies that bind to human HNMT and facilitate the specific and sensitive detection of the protein on immunoblots of human lysates and by immunohistochemical staining of human tissues [10]. Antibody clones HYB372-04/-05/-06/-07/-08/-09 resulted from immunization with human HNMT and clones HYB373-02/-03 resulted from immunization with porcine HNMT but cross-reacted with human HNMT [10]. To analyse where on the human HNMT protein these eight antibodies bind, a series of C-terminal deletions of the 292 amino acid HNMT protein was constructed by recombinant DNA technology and the resulting polypeptides were expressed as GST fusions in bacteria (Fig. 1a; Table 1). Bacterial lysates containing considerable amounts of the respective fusion proteins (Fig. 1b) were then separated by SDS polyacrylamide gel electrophoresis and blotted onto PVDF membranes to test the binding of the antibodies.

**Fig. 2:**
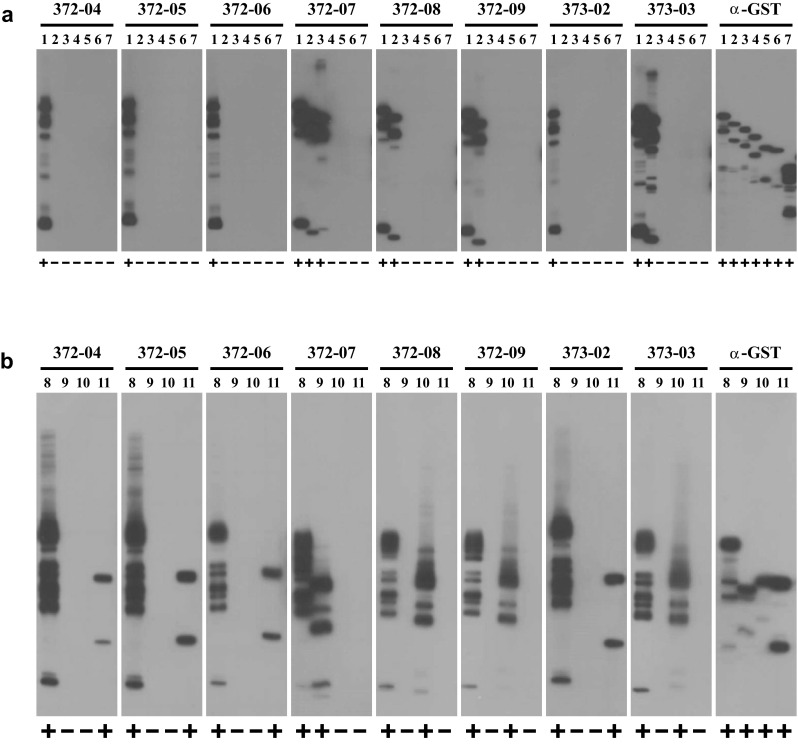
Antibody binding to HNMT fragments. Antibody binding was tested using filter strips of HNMT fragments with C-terminal deletions (**a**) or of HNMT fragments from the C-terminal region (**b**). Filter strips containing approximately 5 µg of cell lysate protein separated on a 12.5% SDS polyacrylamide gel were incubated with the mouse monoclonal antibodies HYB372-04/-05/-06/-07/-08/-09, HYB373-02/-03 (diluted 1:1000–1:20000 in TBSTM), and the anti-GST antibody HYB374-01 (1:1500), respectively. Filter strips were then incubated with horseradish peroxidase-conjugated anti-mouse immunoglobulins (1:1500 in TBSTM), followed by ECL substrate, and exposure to film for 0.25–10 min. *Lane numbers* *1–7* and* 8–11* correspond to expression constructs pGEX-huHMT01-07 and pGEX-huHMT08-11, respectively, and binding is indicated by *plus* and *minus signs below each lane*. Exact positions of the bands on parallel lanes vary slightly because filter strips from different individual blots were used for this experiment. Besides the major band of the respective full-length fusion protein, a variable number of smaller bands are visible in most lanes due to production of partial products in the bacterial expression system

As expected and shown in Fig. 2a, all antibodies gave a strong signal with the GST-HNMT fusion protein containing full-length 292 amino acid human HNMT expressed by plasmid pGEX-huHMT01. Antibodies HYB372-04/-05/-06 and HYB373-02 did not bind any of the shorter fusion proteins indicating that their binding requires residues downstream of *Bgl*II^792^ or D^265^, respectively. Antibodies HYB372-08/-09 and HYB373-03 also bound the fusion protein produced by pGEX-huHMT02 but none of the shorter fragments, indicating that binding requires residues downstream of *Nco*I^668^ or M^224^, respectively. Antibody HYB372-07 also bound the fusion protein produced by pGEX-huHMT03 but none of the shorter fragments, indicating binding requires residues downstream of *Kpn*I^491^ or N^165^, respectively.

The fact that an antibody does not bind a fusion protein lacking a certain C-terminal fragment could either mean that the complete binding site is located in the missing region or that part of the missing region is necessary for forming a proper binding site or conformation with a region further upstream. Therefore, we next constructed clones expressing fragments of the C-terminal region of HNMT as GST fusions and tested these for antibody binding (Fig. 1; Table 1). As shown in Fig. 2b, all antibodies bound the GST fusion of the HNMT peptide N^165^–A^292^ expressed from plasmid pGEX-huHMT08 confirming that their binding sites are indeed located in that C-terminal region of the protein. When testing shorter non-overlapping fragments from this region, we found that HYB372-07 bound peptide L^182^–T^223^ expressed from pGEX-huHMT09, HYB372-08/-09 and HYB373-03 bound peptide M^224^–E^261^ expressed from pGEX-huHMT10, and HYB372-04/-05/-06 and HYB373-02 bound peptide L^262^–A^292^ expressed from pGEX-huHMT11 (Fig. 2b). Thus, the binding sites of all antibodies could be localized on specific small peptides in the C-terminal region of HNMT and appeared to be linear epitopes.

**Fig. 3:**
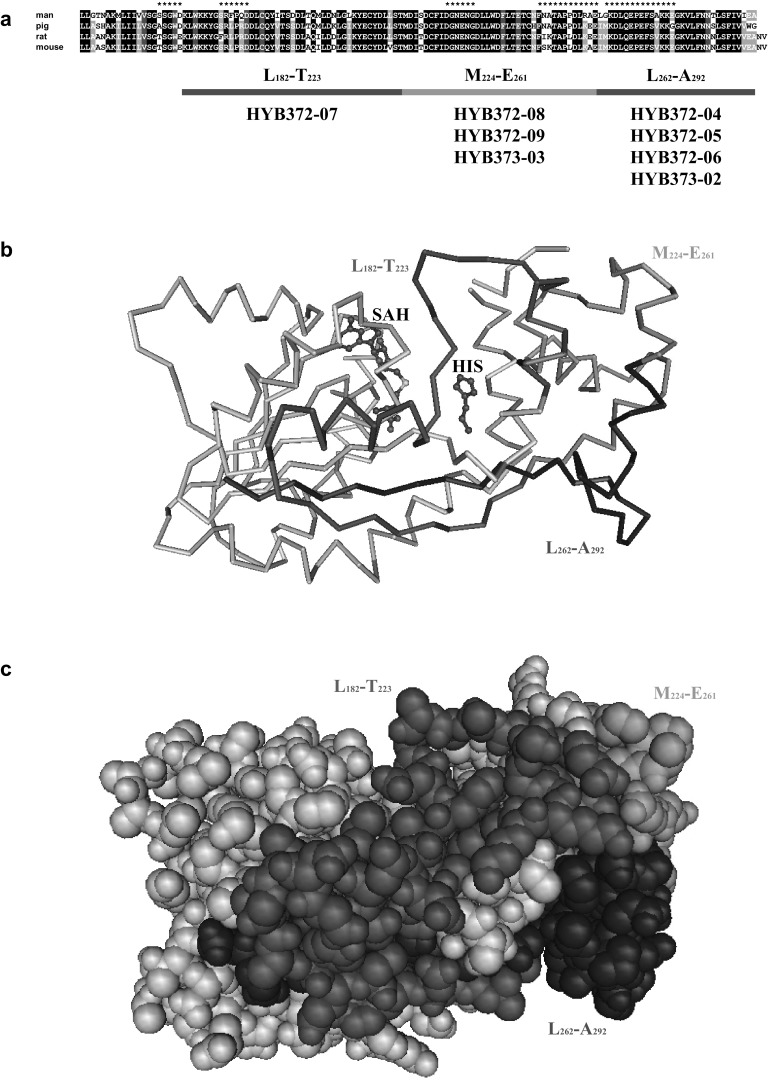
Localization of antibody binding sites. **a** Alignment of human, pig, rat and mouse HNMT polypeptide sequences of the C-terminal domain L^161^–A^292^ with predicted linear epitopes indicated by *asterisk on top*. Residues identical in all four proteins are *shaded black*, residues identical in three proteins are *shaded gray*, and residues that have been shown to interact with histamine in human HNMT [8] are *shaded red*, respectively. Peptide fragments used for testing antibody binding are shown below the sequence with the antibodies binding to them. **b** Protein backbone model and **c** space filling model of human HNMT structure 1JQD [8] with the methyltransferase fold to the *left* and the antibody binding peptide residues L^182^–T^223^, M^224^–E^261^, and L^262^–A^292^ highlighted in *red, green,* and *blue*, respectively. Histamine (HIS) and *S*-adenosyl-l-homocysteine (SAH) bound to HNMT are visible as ball-and-stick models in **b** but are hidden by the polypeptide chain in **c** (color figure online)

HYB372-07 produced strong HNMT specific bands on blots of human, pig, and rat kidney lysates of comparable HNMT enzymatic activity but hardly any signal with a mouse kidney lysate (Table 2). Therefore, this antibody should bind a peptide that is nearly identical in man, pig, and rat but different in mouse. In the HYB372-07 binding region L^182^–T^223^ only the C-terminal peptide K^214^–T^223^ meets this requirement with identical sequences in all species except for position 221 where a leucine in human, pig, and rat HNMT is replaced by a valine in mouse HNMT (Fig. 3a). Moreover, this peptide appears not to be completely exposed on the protein surface (Fig. 3b, c), which might explain the fact that HYB372-07 does not immunoprecipitate native human or porcine HNMT (Table 2).

**Table 2:** Properties of human HNMT specific monoclonal antibodies

Antibody	Isotype	huHNMT	piHNMT	raHNMT	moHNMT	IP_hu_	IP_pi_	BR
HYB372-07	IgG1κ	++	++	++	+/−	−	−	L^182^–T^223^
HYB372-08	IgG1κ	++	++	++	+	+	+	M^224^–E^261^
HYB372-09	IgG1κ	++	++	++	+	+	+	M^224^–E^261^
HYB373-03	IgG1κ	++	++	++	+	+	+	M^224^–E^261^
HYB372-04	IgG1κ	++	−	−	−	++	−	L^262^–A^292^
HYB372-05	IgG1κ	++	−	−	−	++	−	L^262^–A^292^
HYB372-06	IgG1κ	++	−	−	−	++	−	L^262^–A^292^
HYB373-02	IgG1κ	+	+	−	−	−	−	L^262^–A^292^

Species cross-reactivity was tested on blots of human (hu), pig (pi), rat (ra), and mouse (mo) kidney lysates that had comparable HNMT enzymatic activity. Immunoprecipitation of HNMT was tested with human (IP_hu_) and porcine (IP_pi_) kidney and liver lysates, respectively. The binding region (BR) specifies the peptide of human HNMT recognized on blots

++, strong; +, weak; −, no binding or immunoprecipitation

HYB372-08/-09 and HYB372-02, which bound in region M^224^–E^261^, also produced strong HNMT bands on blots of human and pig kidney lysates, slightly weaker bands with a rat lysate, and very weak bands with a mouse lysate but partially immunoprecipitated native human and pig HNMT (Table 2). Thus, these antibodies should bind a peptide that is nearly identical in man and pig, slightly different in rat and significantly different in mouse. Only the C-terminal peptide T^247^–E^261^ of region M^224^–E^261^ contains variant sequences starting at N^249^ and including a notable change of N^251^A^252^ present in human and pig HNMT to IK in rat and SK in mouse HNMT. This peptide has been predicted as a likely epitope in human and pig HNMT [19] and is accessible on the surface of the HNMT protein in accordance with the immunoprecipitation results (Fig. 3a–c).

HYB372-04/-05/-06, which bound in region L^262^–A^292^, produced strong HNMT bands only on blots of human kidney lysates but did not react with HNMT from other species and exhibited strong immunoprecipitation of native human HNMT (Table 2). Therefore, these antibodies should bind a peptide on the surface of HNMT with distinct sequence differences in the human protein. The peptide L^262^–E^276^ predicted as a likely epitope [19] contains residues L^262^, G^263^, and A^273^ in human HNMT that are different in all other HNMT sequences (Fig. 3a) and forms a well-exposed loop on the surface of the protein (Fig. 3b, c). In contrast, HYB373-02 binding in region L^262^–A^292^ produced only weak HNMT bands on blots of human and pig kidney lysates, did not react with rat and mouse HNMT, and did not immunoprecipitate human or pig HNMT (Table 2). Accordingly, this antibody probably binds peptide G^277^–V^289^ that contains a conservative substitution of T^284^ in human HNMT to S^284^ in pig HNMT but a non-conservative substitution to N in rat and mouse HNMT (Fig. 3a) and is not accessible on the surface of the native protein (Fig. 3b, c).

## Discussion

The binding sites of eight mouse monoclonal antibodies specific for human HNMT [10] were determined by antibody binding on blots of HNMT protein fragments expressed in vitro combined with information from species cross-reactivity, sequence comparison, protein structure, and binding site prediction software. With this approach binding of the antibodies could be mapped to relatively short linear peptide fragments in the C-terminal domain of HNMT including K^214^–T^223^ for HYB372-07, T^247^–E^261^ for HYB372-08/-09 and HYB373-03, L^262^–E^276^ for HYB372-04/-05/-06, and G^277^–V^289^ for HYB373-02. Although we did not map the exact epitopes using specific peptides and thus the actual binding sites might be slightly larger or smaller than designated above, the position information is adequate to state that different antibodies bind to separate, non-overlapping regions and to explain the different species cross-reactivity of the antibodies and the surface accessibility of each binding site.

These antibodies were obtained by immunization of mice with recombinant full-length human and porcine HNMT, respectively, that had full enzymatic activity, and therefore, probably was in their native conformation, and initial screening was done by ELISA using the native proteins as antigens [10]. Whereas all antibodies exhibited excellent binding to denatured HNMT on Western blots and in immunohistochemistry, only HYB372-04/-05/-06/-08/-09 and HYB373-03 were able to immunoprecipitate enzymatically active HNMT from human kidney and liver lysates, indicating that the epitopes recognized by these antibodies are accessible on the surface of the native protein (Table 2; Fig. 3b, c). In contrast, HYB372-07 and HYB373-02 did not immunoprecipitate native human HNMT, indicating a limited accessibility of their respective epitopes on the native protein. Interestingly, the putative binding regions of the antibodies immunoprecipitating HNMT were accurately predicted by the binding site prediction software [19] whereas those of the antibodies not immunoprecipitating HNMT were not (Fig. 3a; Table 2).

HYB372-04/-05/-06 bound only to human HNMT but not to pig, rat, and mouse HNMT and HYB373-02 bound only to human and pig HNMT but not to rat and mouse HNMT, which is probably a consequence of distinct sequence differences in the putative binding regions (Fig. 3a). So these antibodies will be useful only for analyses of the human and porcine HNMT proteins, respectively. On the other hand, HYB372-07/-08/-09 and HYB373-03 exhibited strong binding to human, pig, and rat HNMT and very weak binding to mouse HNMT (Table 2), which can be explained by the sequence conservation of the putative binding regions in different species and small but significant sequence differences in mice (Fig. 3a). As the respective HNMT sequences are conserved in many other species besides those tested here, these antibodies will also be useful for studies of the HNMT protein in other model organisms.

In conclusion, the eight HNMT monoclonal antibodies bound in three different regions of the protein and those binding the same putative epitope exhibited similar binding characteristics and species cross-reactivity. Having a collection of antibodies binding separate non-overlapping epitopes will facilitate the comprehensive analysis of native human HNMT but also of all HNMT variants resulting from altered HNMT gene sequences. Our binding studies with partial HNMT peptide fragments showed that the antibodies will bind to any HNMT variant that contains an unaltered binding sequence. The antibodies will be especially useful for the study of SNPs leading to amino acid substitutions associated with altered enzyme function that might be relevant for various human diseases such as the T^105^I substitution associated with reduced enzyme activity and stability [11] as well as the G^60^D and the L^208^P substitutions identified in patients with intellectual disability [12].

## Abbreviations

DAO: Diamine oxidase
GST: Glutathione *S*-transferase
HDC: Histidine decarboxylase
HNMT: Histamine *N*-methyltransferase
SNP: Single nucleotide polymorphism
